# Supplementary material for: Cryo-EM structure of the CDK2-cyclin A-CDC25A complex
Source: Nat Commun. 2024 Aug 9;15:6807. doi: 10.1038/s41467-024-51135-w (PMC11316097; doi:10.1038/s41467-024-51135-w)
Supplement: Supplementary file 3 — Reporting summary [file 41467_2024_51135_MOESM3_ESM.pdf]

nature portfolio

Corresponding author(s): Jane A. Endicott, Martin E. M. Noble

Last updated by author(s): Jun 24, 2024

Reporting Summary

Nature Portfolio wishes to improve the reproducibility of the work that we publish. This form provides structure for consistency and transparency in reporting. For further information on Nature Portfolio policies, see our Editorial Policies and the Editorial Policy Checklist.

Statistics

For all statistical analyses, confirm that the following items are present in the figure legend, table legend, main text, or Methods section.

n/a	Confirmed	
☐	☒	The exact sample size (*n*) for each experimental group/condition, given as a discrete number and unit of measurement
☐	☒	A statement on whether measurements were taken from distinct samples or whether the same sample was measured repeatedly
☒	☐	The statistical test(s) used AND whether they are one- or two-sided<br>*Only common tests should be described solely by name; describe more complex techniques in the Methods section.*
☒	☐	A description of all covariates tested
☒	☐	A description of any assumptions or corrections, such as tests of normality and adjustment for multiple comparisons
☒	☐	A full description of the statistical parameters including central tendency (e.g. means) or other basic estimates (e.g. regression coefficient) AND variation (e.g. standard deviation) or associated estimates of uncertainty (e.g. confidence intervals)
☒	☐	For null hypothesis testing, the test statistic (e.g. *F*, *t*, *r*) with confidence intervals, effect sizes, degrees of freedom and *P* value noted<br>*Give P values as exact values whenever suitable.*
☒	☐	For Bayesian analysis, information on the choice of priors and Markov chain Monte Carlo settings
☒	☐	For hierarchical and complex designs, identification of the appropriate level for tests and full reporting of outcomes
☒	☐	Estimates of effect sizes (e.g. Cohen's *d*, Pearson's *r*), indicating how they were calculated

Our web collection on statistics for biologists contains articles on many of the points above.

Software and code

Policy information about availability of computer code

Data collection	Cryo-EM data collection was performed using the ThermoFisher EPU software.
Data analysis	CryoSPARC, UCSF ChimeraX (v1.7), Phenix (v.1.20.1), Model Angelo, Coot, QtPISA (v2.1.0), AlphaFold2 Multimer accessed via ColabFold (v1.5.5), Cryo-EF, GraphPad Prism 6, PLGS (v3.0.2) and DynamX (v3.0.0) (both Waters), Deuteros 2.0 (https://github.com/andymlau/Deuteros_2.0), Biacore S200 evaluation software (v1.0)(Cytiva). All structural figures were generated using UCSF ChimeraX (v1.7).

For manuscripts utilizing custom algorithms or software that are central to the research but not yet described in published literature, software must be made available to editors and reviewers. We strongly encourage code deposition in a community repository (e.g. GitHub). See the Nature Portfolio guidelines for submitting code & software for further information.

Data

Policy information about availability of data

All manuscripts must include a data availability statement. This statement should provide the following information, where applicable:

- Accession codes, unique identifiers, or web links for publicly available datasets
- A description of any restrictions on data availability
- For clinical datasets or third party data, please ensure that the statement adheres to our policy

The cryo-EM map and model of the trimeric CDK2-cyclin A-CDC25A complex generated in this study have been deposited in the Protein Data Bank and the Electron Microscopy Data Bank with accession numbers PDB 8ROZ [https://doi.org/10.2210/pdb8ROZ/pdb] and EMD-19408 [https://www.ebi.ac.uk/emdb/EMD-19408]. The

mass spectrometry proteomics data have been deposited to the ProteomeXchange Consortium via the PRIDE86 partner repository with the dataset identifier PXD050866 [https://www.ebi.ac.uk/pride/archive/projects/PXD050866]. The Surface Plasmon Resonance and the Homogeneous Time-Resolved Fluorescence data generated in this study are provided in the Supplementary Information. Source data are provided with this paper in the Source Data file.

Research involving human participants, their data, or biological material

Policy information about studies with human participants or human data. See also policy information about sex, gender (identity/presentation), and sexual orientation and race, ethnicity and racism.

Reporting on sex and gender	Not applicable, no human subjects were involved in this study
Reporting on race, ethnicity, or other socially relevant groupings	Not applicable, no human subjects were involved in this study
Population characteristics	Not applicable, no human subjects were involved in this study
Recruitment	Not applicable, no human subjects were involved in this study
Ethics oversight	Not applicable, no human subjects were involved in this study

Note that full information on the approval of the study protocol must also be provided in the manuscript.

Field-specific reporting

Please select the one below that is the best fit for your research. If you are not sure, read the appropriate sections before making your selection.

☒ Life sciences ☐ Behavioural & social sciences ☐ Ecological, evolutionary & environmental sciences

For a reference copy of the document with all sections, see nature.com/documents/nr-reporting-summary-flat.pdf

Life sciences study design

All studies must disclose on these points even when the disclosure is negative.

Sample size	No sample size calculations were performed. Protein solutions at appropriate concentrations were analysed by cryo-EM and biophysical techniques (HDX-MS, SPR and HTRF). Cryo-EM data were collected until a suitable structural solution was achieved.
Data exclusions	During cryo-EM data collection and processing, poor quality micrographs were excluded (informed by CTF fit, total motion and ice quality) and poor particles were subsequently removed during 2D and 3D classification. There were no data exclusions for the SPR and HTRF studies. HDX-MS data were manually curated and data were excluded in cases where individual spectra had poor signal-to-noise.
Replication	For cryo-EM studies, initial screening across multiple grids was performed to identify the best grids for high resolution data collection. Consistent high resolution data was collected over 2 grids. Number of experimental replicates for HTRF were at least 2 biological replicates each run in triplicate and carried out on separate days. SPR experiments were single replicates of 7-point dilution series. HDX-MS was carried out in 3 technical replicates. Details are included in the experimental methods and supplementary information.
Randomization	Sample randomization does not apply to this study. Protein solutions at appropriate concentrations were analysed by cryo-EM and biophysical techniques (HDX-MS, SPR and HTRF). Covariate analysis of the experiments performed is not applicable.
Blinding	Cryo EM in this study did not generate comparative data for differing samples so that blinding is not relevant. Sample presentation and data analysis in HDX, SPR and HTRF are automated to the point that cognitive biases cannot impact the results that arise.

Reporting for specific materials, systems and methods

We require information from authors about some types of materials, experimental systems and methods used in many studies. Here, indicate whether each material, system or method listed is relevant to your study. If you are not sure if a list item applies to your research, read the appropriate section before selecting a response.

Materials & experimental systems

n/a	Involved in the study	
☐	☒	Antibodies
☒	☐	Eukaryotic cell lines
☒	☐	Palaeontology and archaeology
☒	☐	Animals and other organisms
☒	☐	Clinical data
☒	☐	Dual use research of concern
☒	☐	Plants

Methods

n/a	Involved in the study	
☒	☐	ChIP-seq
☒	☐	Flow cytometry
☒	☐	MRI-based neuroimaging

Antibodies

Antibodies used	Polyclonal goat anti-GST antibody, purchased from Cytiva (BR100223).<br>HTRF monoclonal antibody Anti GST-Tb cryptate, purchased from Revitty (61GSTTLB)
Validation	Antibodies sourced commercially.

Plants

Seed stocks	Not applicable, no plants used in this study
Novel plant genotypes	Not applicable, no plants used in this study
Authentication	Not applicable, no plants used in this study
